# Supplementary material for: Gene expression in cord blood links genetic risk for neurodevelopmental disorders with maternal psychological distress and adverse childhood outcomes
Source: Brain Behav Immun. 2018 Oct;73:320–30. doi: 10.1016/j.bbi.2018.05.016 (PMC6191930; doi:10.1016/j.bbi.2018.05.016)
Supplement: Supplementary data 2 [file mmc2.pdf]

SUPPLEMENTARY FIGURES

Title: Gene expression in cord blood links genetic risk for neurodevelopmental disorders with maternal psychological distress and adverse childhood outcomes

Authors: Michael S. Breen[1,2,3*], Aliza P. Wingo[4,5], Nastassja Koen[6], Kirsten A. Donald[6,7], Mark Nicol[8], Heather J. Zar[7], Kerry J. Ressler[5,9], Joseph D. Buxbaum[1,2,3], Dan J. Stein[6*]

Affiliations: [1]Department of Psychiatry, Icahn School of Medicine at Mount Sinai, New York, New York, USA; [2]Department of Genetic and Genomic Sciences, Icahn School of Medicine at Mount Sinai, New York, New York, USA; [3]Seaver Autism Center for Research and Treatment, Icahn School of Medicine at Mount Sinai, New York, New York, USA; [4]Atlanta Veterans Affairs Medical Center, Atlanta, Georgia, USA; [5]Department of Psychiatry, School of Medicine, Emory University, Atlanta, Georgia, USA; [6]Department of Psychiatry and Mental Health, University of Cape Town South Africa; and South African Medical Research Council (SAMRC) Unit on Child and Adolescent Health, University of Cape Town, Cape Town, South Africa; [7]Department of Paediatrics and Child Health and MRC Unit on Child & Adolescent Health, University of Cape Town, Cape Town, South Africa; [8]Division of Medical Microbiology, Department of Pathology, University of Cape Town and National Health Laboratory Service, South Africa; [9]McLean Hospital, Harvard Medical School, Belmont, Massachusetts, USA.

Correspondence to: michael.breen@mssm.edu and dan.stein@uct.ac.za

This file contains **Supplementary Figures 1-8**. In brief:

Supplementary Figure 1. Gene expression quality control and cell type frequency estimates.
Supplementary Figure 2. Differential gene expression signatures and associations.
Supplementary Figure 3. WGCNA network analysis.
Supplementary Figure 4. Prenatal depression co-expression modules M2 and M13.
Supplementary Figure 5. Gene significance and kME associations for disease modules.
Supplementary Figure 6. Prenatal PTSD co-expression modules M3 and M14.
Supplementary Figure 7. Distribution of genetic risk loci throughout intramodular connectivity.
Supplementary Figure 8. Power and sample size estimates.

Please see Supplementary Table file for Supplementary Tables 1-7.

Figure S1

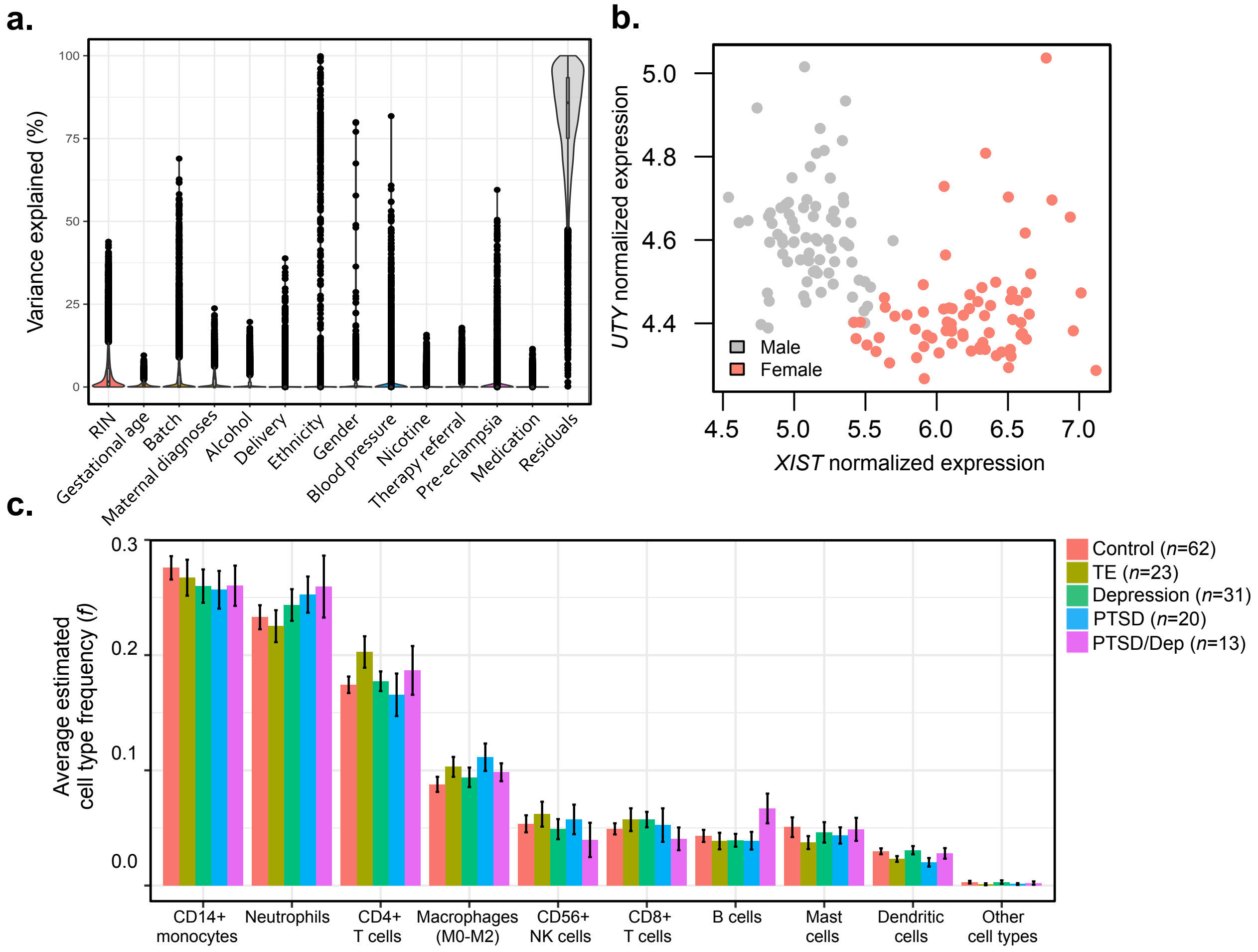

Supplementary Figure 1. Quality control and cell type estimates for all gene expression samples (149) passing outlier removal. (**a**) variancePartition analysis of global cord blood gene expression profiles identifies RNA integrity numbers (RIN) as a leading trait explain the most amount of observed gene expression variability (~3%), followed by batch, alcohol and mode of delivery. (**b**) Scatterplot of normalized XIST and UTY gene expression to confirm neonate gender. (**c**) Cibersort cell type deconvolution analysis of global cord blood gene expression profiles estimated cell frequencies (y-axis) for 9 major immune cell types and 13 less frequent immune cell subsets pooled as 'other' (x-axis). Standard error bars are presented. Each estimate partitioned by prenatal exposure (*e.g.* PTSD, TE, depression *etc*…). A Dunnet's multiple comparison of means test was used to assess differences between each prenatal exposure group with neonates born to healthy mothers and comparatively equal numbers of estimated cell type frequencies were found across all groups. Abbreviations: PregMed, current medication; PrevMed, previous medication.

Figure S2

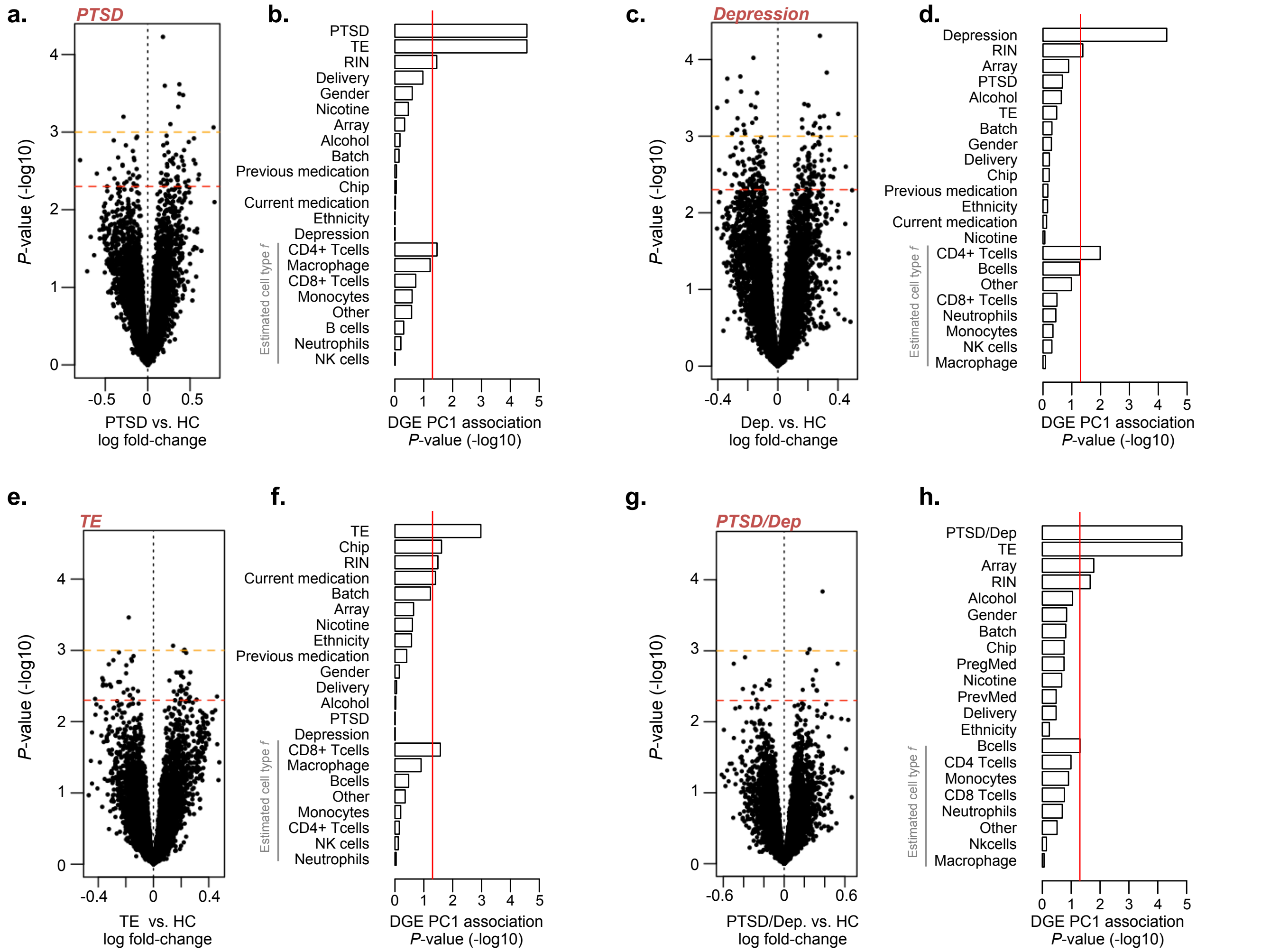

Supplementary Figure 2. Differential gene expression (DGE) analyses and influence of covariates on detected signatures. Volcano plot comparing extent of $\log_2$ fold-change to -log10 *P*-value significance for DGE associated to prenatal maternal (**a**) PTSD, (**c**) depression (**e**) TE and (**g**) PTSD/Dep. Red dotted line indicates genes passing P<0.005 and orange dotted line indicates genes passing P<0.001. To quantify the influence of covariates on all DGE signatures, we computed the first principal component (PC1) for all genes passing a suggestive *P*-value threshold (P<0.05) and correlated this measure to all available covariates for all comparisons, including (**b**) PTSD, (**d**) depression, (**f**) TE and (**h**) PTSD/Dep.

Figure S3

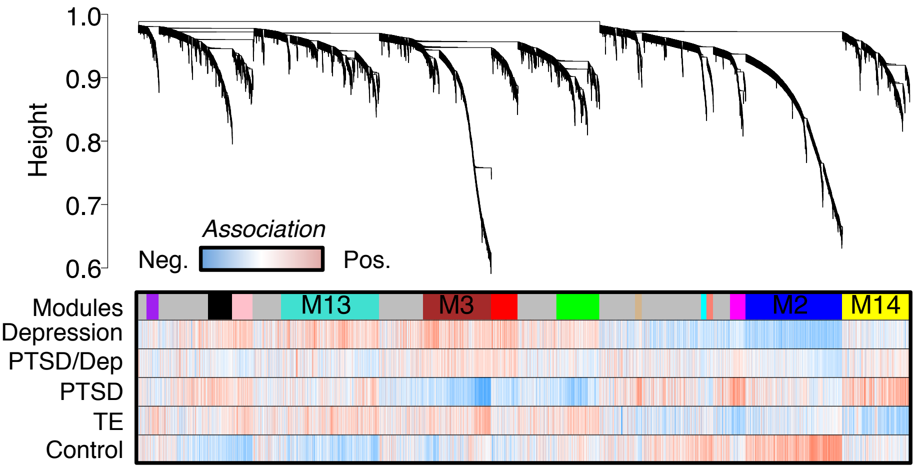

Supplementary Figure 3. Weighted gene co-expression network analysis of 149 cord blood transcriptome profiles (n^{genes}=10,705). Hierarchical gene cluster tree and module structure and gene-phenotype color bands. The first color band underneath the tree indicates the fourteen detected modules and subsequent bands indicate gene-phenotype correlation, when red indicates a strong relationship and blue indicates a strong negative relationship.

Figure S4

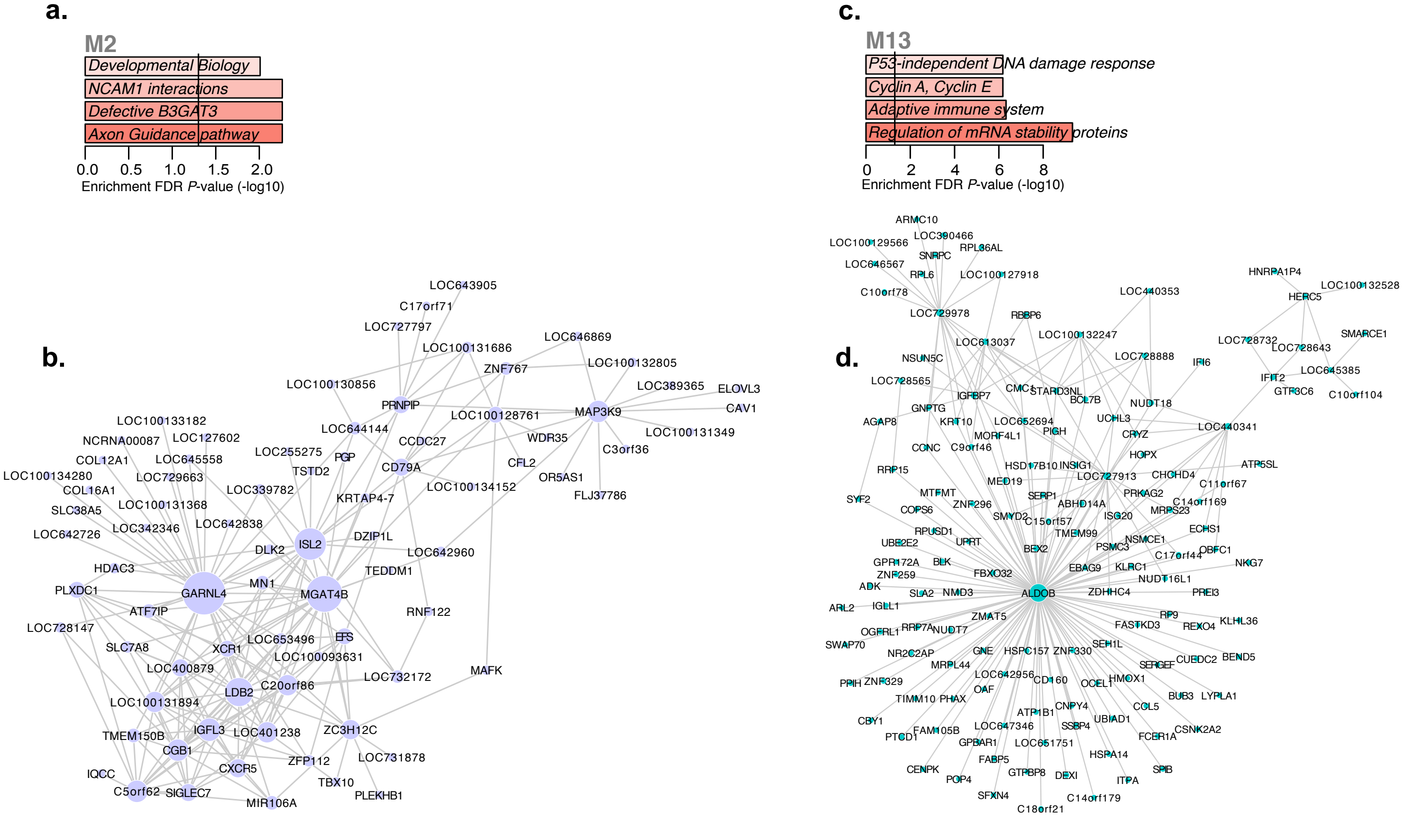

Supplementary Figure 4. Module characterization for prenatal exposure to maternal depression. Enrichment analysis and co-expression networks for module (**a-b**) M2 and (**c-d**) module M13. Enrichment analysis was used to identify the top REACTOME ontology terms within each module. Co-expression networks were constructed selecting the top 200 most significant associations ranked by kME. Nodes represent genes and edges represent correlations. Hub genes are those more correlated to ME values and predicted to be of importance for module function, and are shown in larger sizes.

Figure S5

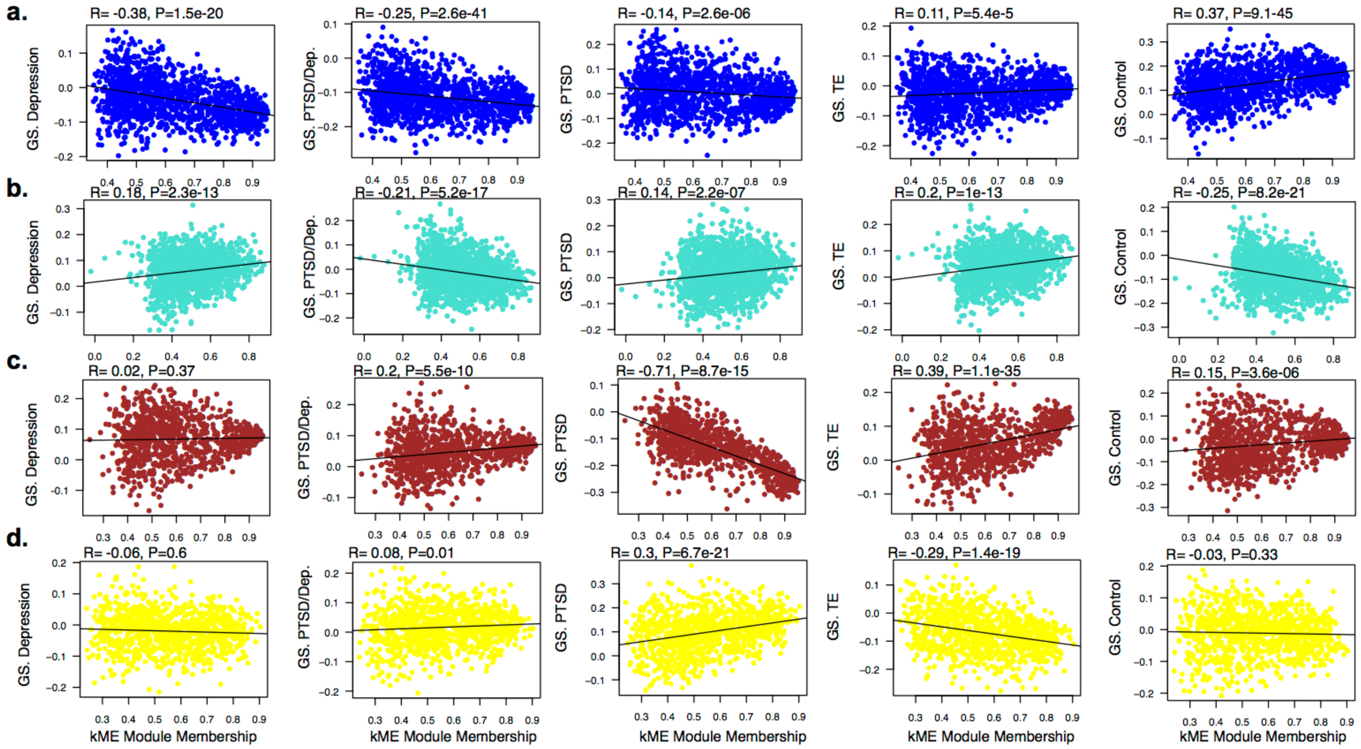

Supplementary Figure 5. Scatterplots of intramodular connectivity (kME) compared to gene significance (GS). Scatterplots were created for modules of interest, including modules (**a**) M2 in blue, (**b**) M13 in turquoise, (**c**) M3 in brown and (**d**) M14 in yellow. kME measures how connected, or co-expressed, a given gene is with respect to the genes of a particular module. The intramodular connectivity may be interpreted as a measure of module membership. Meanwhile, the higher the absolute value of gene significance $(GS)_i$, the more biologically significant is the *i*-th gene. GS was computed measuring correlation to dummy coded phenotype label (*i.e.* 1 or 0).

Figure S6

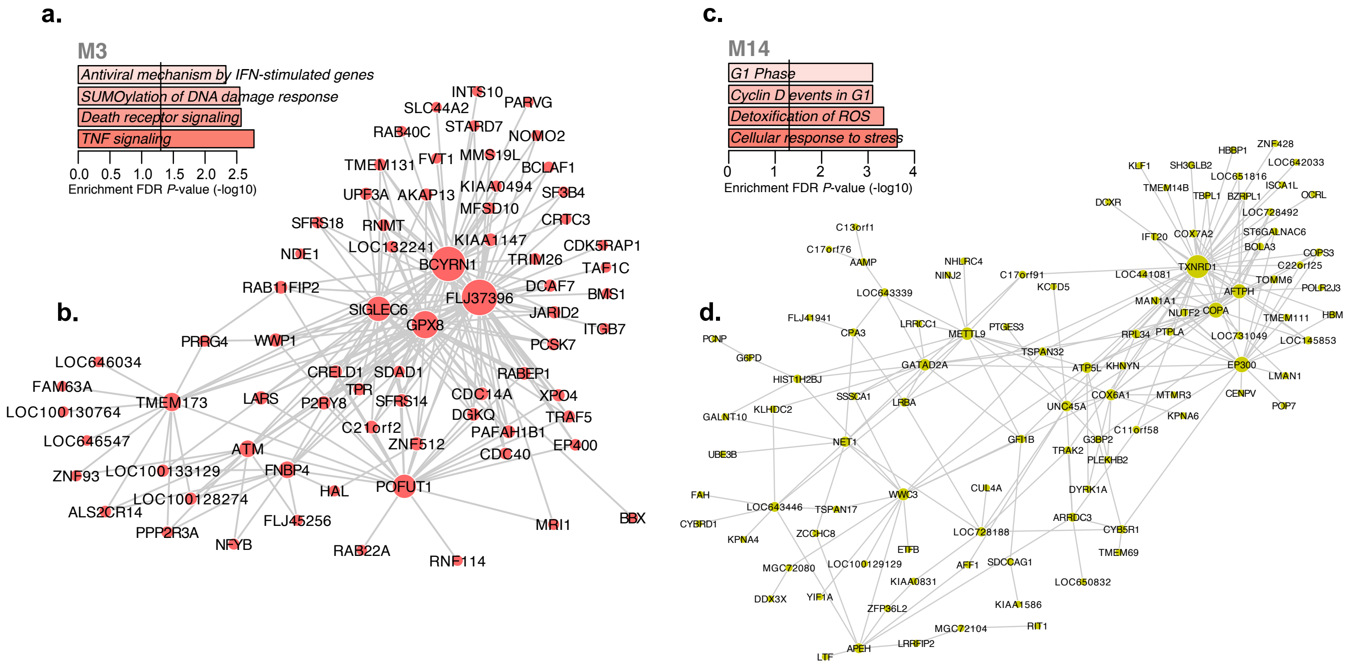

Supplementary Figure 6. Module characterization for prenatal exposure to maternal PTSD. Enrichment analysis and co-expression networks for module (**a-b**) M3 and (**c-d**) module M14. Enrichment analysis was used to identify the top Reactome ontology terms within each module. Co-expression networks were constructed selecting the top 200 most significant associations ranked by kME. Nodes represent genes and edges represent correlations. Hub genes are those more correlated to ME values and predicted to be of importance for module function, and are shown in larger sizes.

Figure S7

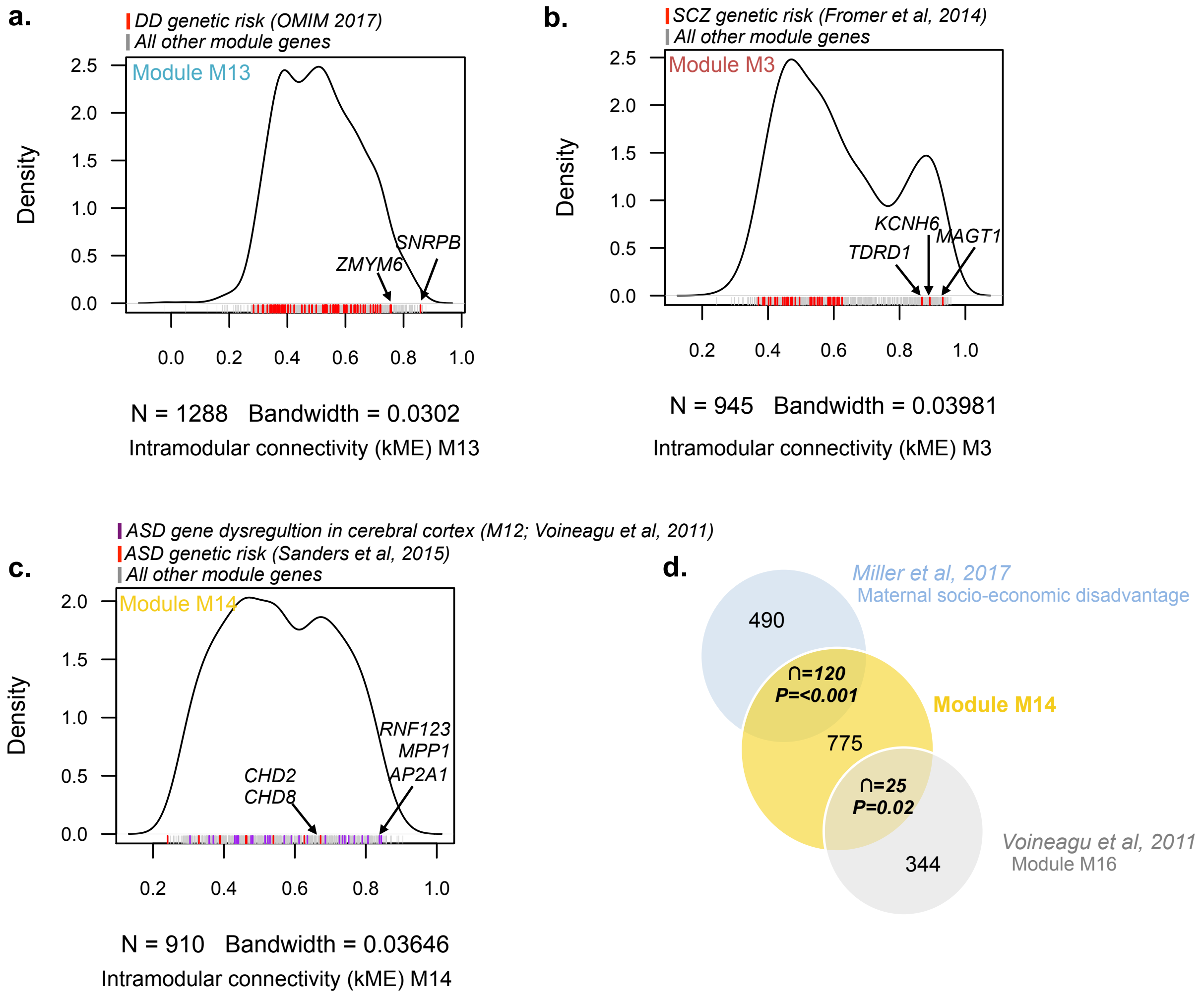

Supplementary Figure 7. Distribution of genetic risk loci across intramodular connectivity (kME). Distribution of genetic risk variants for (**a**) developmental delay (DD) in module M13, (**b**) schizophrenia (SCZ) in module M3 and (**c**) autism spectrum disorder (ASD) in module M14. Histograms of kME with rug plots indicating the presence of genetic variants for neurodevelopmental disorders (red) are plotted along the x-axis. (**d**) Over-representation analyses of dysregulated genes previously identified *i*) in umbilical cord blood from neonates born to mothers with socio-economic disadvantage (Miller et al., 2017) and *ii*) in the cerebral cortex of ASD cases (Voineagu *et al.,* 2011). Dysregulated genes identified by Miller et al., 2017 were implicated in activated immune response, while dysregulated module M16 identified by Voineagu et al., 2011 was implicated in innate immune and inflammatory function.

Figure S8

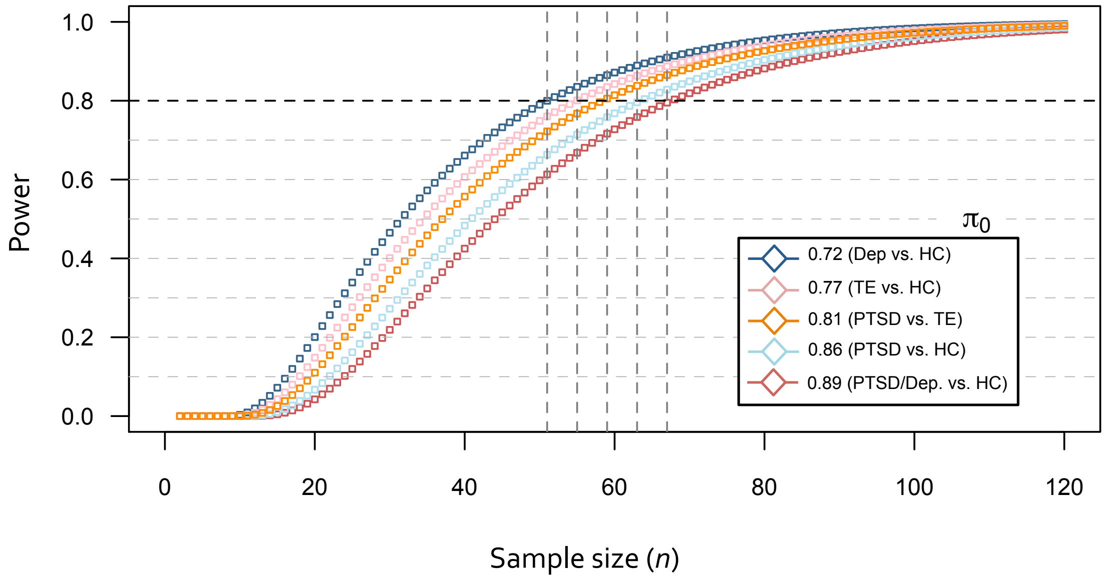

Supplementary Figure 8. Power and sample size estimates based on differential gene expression (DGE). In our dataset, Δ (the common difference in mean expression between the two group samples for all genes) =0.89, Σ (the common standard deviation of expressions for all genes) = 1.5 and pi0 (the proportion of non-differentially expressed genes for each comparison) are displayed in the inset figure. In order to reach a power of 80%, a sample size of 51 is needed for maternal depression, 55 for maternal TE, 59 for maternal PTSD (when compared to TE), 63 for PTSD (when compared to HC) and 67 for PTSD/Dep. Sample size refers to the number of cases and the number of controls needed.
